# Disabling pelvic pain following open surgery for rectal prolapse: a case report

**DOI:** 10.4076/1752-1947-3-9214

**Published:** 2009-09-15

**Authors:** Sébastien Romy, Maurice JC Matter, Christian Felley, Nicolas Demartines

**Affiliations:** 1 Service de Chirurgie Viscérale, Centre Hospitalier Universitaire Vaudois, 1011 Lausanne, Suisse; 2 Service de Gastroentérologie, Centre Hospitalier Universitaire Vaudois, 1011 Lausanne, Suisse

## Abstract

**Introduction:**

Iatrogenic inferior hypogastric plexus neuropathy is a well-reported side effect of rectal prolapse surgery. This case report emphasizes the importance of careful evaluation of surgical strategy in pelvic surgery.

**Case presentation:**

A 60-year-old Swiss Caucasian woman developed disabling pelvic pain in the right iliac fossa, radiating to the upper posterior side of the right thigh and right labium majus characterized by electric feelings. This followed resection and bilateral rectal fixation to the sacral promontory as treatment for rectal prolapse. Investigations included a multidisciplinary neurological pain evaluation. A computed tomography scan did not reveal any cause. Revision surgery was performed and a foreign body, a thread, was found wrapped around the inferior hypogastric plexus and was removed. Four years later, the patient remains asymptomatic.

**Conclusion:**

This case emphasizes the importance of careful identification of the inferior hypogastric plexus during primary pelvic surgery.

## Introduction

Post-operative disabling chronic pelvic pain with sexual dysfunction in women due to iatrogenic inferior hypogastric plexus (IHP) neuropathy is a well-reported side effect of pelvic surgery, described mainly after hysterectomy but also following total mesorectal resection and rectal prolapse operations [1]-[3].

## Case presentation

**Figure 1:**
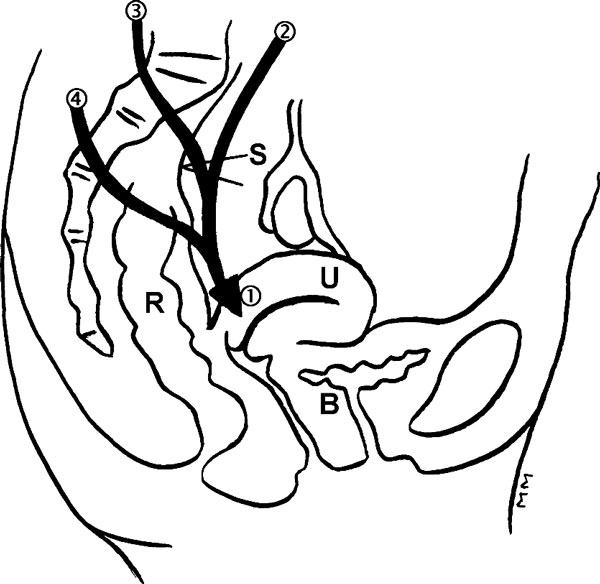
**Innervation of the pelvis and position of the stitch (S) with two possible explanations: direct contact with inferior hypogastric plexus and direct trauma with the tips of the polypropylene stitch**. 1. Inferior hypogastric plexus. 2. Superior hypogastric plexus. 3. Sacral splanchnic nerves. 4. Pelvic slanchnic nerves. R: Rectum. U: Uterus. B: Bladder.

A 60-year-old Swiss Caucasian woman suffering from depression was referred for chronic pelvic pain. Her medical history revealed that a hysterectomy for benign disease had been performed 17 years previously and a laparotomy and surgical repair had been carried out for rectal prolapse two years previously. That procedure included sigmoid resection and bilateral rectal fixation to the sacral promontory with polypropylene thread. Her hospital stay was uneventful but early increasing pain in the right iliac fossa, radiating to the upper posterior side of the right thigh and right labium majus was reported. The pain was characterized by electric feelings that decreased after defecation. She reported dyspareunia but no urinary complaints. One year later a new laparotomy with adhesiolysis was performed but the pelvic pain remained unchanged. New investigations included a computed tomography scan and a complete multidisciplinary neurological evaluation with abdominal wall lidocaine infiltration allowed exclusion of ilioinguinal or genitofemoral neuropathy. A possible iatrogenic hypogastric plexus neuropathy was considered mainly based on the history and status. Digital rectal examination precisely reproduced the pain on the right lateral side of the proximal rectum. A second laparotomy was performed and a 3.0 polypropylene thread used during the initial surgery was exposed and found to be causing partial entrapment of the inferior hypogastric plexus (Figure 1). The foreign material was removed. Some granulation tissue was described histologically but had no neuroma. Pelvic pain disappeared after the revision surgery. The recovery was uneventful and four years later the patient remains asymptomatic.

## Discussion

Chronic pelvic pain in women is defined as noncyclic pain lasting for 6 months [4] or more and includes several symptoms such as dyspareunia, vulvar pain, dysmenorrhea and complaints of pain in the lower abdomen, pelvic floor, or uterus [5]. Chronic pelvic pain is often associated with psychological disturbances like depression or anxiety [6]. Diagnosis is challenging, often frustrating both the patient and surgeon. Evaluation starts with a careful review of the pain history and a thorough physical examination [7,8].

Treatment options may be medical or surgical. Medical treatments may include hormonal suppression, pain medications, physical therapy or psychotherapy [9]. Some trials suggest that steroids can be useful in relieving symptoms [10]. Progesterone on the other hand is the medical gold standard treatment for endometriosis [11]. Surgical treatments range from diagnostic laparoscopy or neuroablative procedures to partial resection of pelvic organs or hysterectomy. Carter *et al.*[12] reported successful pain reduction in about 80% of women treated by laparoscopy or laparotomy after 3 years of follow-up.

## Conclusion

Our case emphasizes the importance of careful identification of the inferior hypogastric plexus during primary pelvic surgery.

## Abbreviation

IHP: inferior hypogastric plexus.

## Competing interests

The authors declare that they have no competing interests.

## Consent

Written informed consent was obtained from the patient for publication of this case report. A copy of the written consent is available for review by the Editor-in-Chief of this journal.

## Authors' contributions

SR conceived the case report. MM was involved in drafting the manuscript and revising it critically. CF made contributions to the acquisition of data. ND was involved in critically revising the manuscript and has given final approval of the version to be published.
